# The *in vitro* stimulatory activity of CwlM∼P on mycobacterial MurA depends on a charged predicted interface that is distal from the CwlM phosphorylation site

**DOI:** 10.3389/fmicb.2025.1543775

**Published:** 2025-12-09

**Authors:** Augusto Cesar Hunt-Serracin, Karen E. Tembiwa, Arinze Awagu, Soroush Ghaffari, Katie N. Kang, Cara C. Boutte

**Affiliations:** 1 Department of Biology, The University of Texas at Arlington, Arlington, TX, United States; 2 Department of Kinesiology, The University of Texas at Arlington, Arlington, TX, United States; 3 Department of Immunology, University of Texas Southwestern Medical Center, Dallas, TX, United States

**Keywords:** peptidoglycan, mycobacteria, protein-protein interactions, enzyme, cell wall

## Abstract

*Mycobacterium tuberculosis* and other mycobacteria have a thick cell wall that is tightly regulated - ensuring cell integrity and promoting antibiotic tolerance. The peptidoglycan layer of the cell wall is controlled at multiple points along the synthesis pathway. Here we focus on the stimulation of the peptidoglycan precursor enzyme MurA by its regulator CwlM. Using AlphaFold2 Multimer predictions we predicted a charged regulatory interface between CwlM and MurA that is separate from the phosphorylation site on CwlM that was previously shown to be required for activation. Using mutants of CwlM, we found that all of CwlM∼P’s stimulation of MurA activity is dependent on this charged interface *in vitro*. However, our *in vitro* results here and previously suggest that MurA may require activators besides CwlM∼P to be fully active. Our *in vivo* studies of the CwlM charged interface mutants corroborate this: we observed no defects in growth and only mild defects in peptidoglycan metabolism. Our findings suggest that MurA regulation by CwlM involves at least two sites on the CwlM protein, and that regulation of MurA *in vivo* is only modestly affected by CwlM, and likely involves other, unknown regulatory factors.

## Introduction

*Mycobacterium tuberculosis* (*Mtb*) is the causative agent of the lung disease tuberculosis (TB) (CDCTB, 2023). *Mtb* infections are challenging to treat partly due to the bacterium’s responses to host stresses, which cause it to fortify its cell envelope, and downregulate many metabolic processes (Baker and Abramovitch, 2018; Chang and Guan, 2021; Dulberger et al., 2019). Although the mycobacterial cell envelope structure is fairly well described (Abrahams and Besra, 2021; Alderwick et al., 2015; Marrakchi et al., 2014; Minnikin, 1991), there are many open questions about its regulation (Dulberger et al., 2020).

The innermost layer of the mycobacterial cell wall is the peptidoglycan, composed of a mesh of glycan strands cross-linked by short peptides (Lederer et al., 1975). The peptidoglycan layer is covalently attached to polysaccharides known as arabinogalactan, which are in turn covalently attached to the inner leaflet of the mycolic acid outer membrane - these layers form the core of the mycobacterial cell wall (Daffe et al., 1990; Minnikin, 1991). Regulation of the cell wall is critical for responding to environmental stress such as those encountered in infection (Arejan et al., 2024; Boutte et al., 2016; Dulberger et al., 2020; Gupta et al., 2022; Hayashi et al., 2018; Melzer et al., 2022; Rego et al., 2017; Shamma et al., 2021). Therefore, understanding this regulation could lead to more effective therapies against mycobacteria.

The synthesis of peptidoglycan begins in the cell’s cytoplasm with the sugar nucleotide UDP-GlcNAc (uridine-diphosphate N-aceytl-glucosamine) which is modified by the enzyme MurA to enoylpyruvyl-UDP-GlcNAc (Marquardt et al., 1992). Subsequent cytoplasmic steps in peptidoglycan precursor synthesis result in lipid II (undecaprenyl-diphosphate-disaccharide-pentapeptide), which is then transported across the inner membrane. The disaccharide-pentapeptide of lipid II is then added to the glycan strands of peptidoglycan by transglycosylases, and cross-linked into the network by transpeptidases.

MurA catalyzes the first committed step of peptidoglycan precursor synthesis and is essential for survival in most bacteria. The MurA structure and enzymatic activity have been described in Gram-negative bacteria, primarily in *Escherichia coli* (*E. coli*) and *Enterobacter cloacae* (Brown Vivas et al., 1995; Funes Chabán et al., 2021; Salton and Kim, 1996; Xu et al., 2014). Some Gram-positive bacteria, like *Staphylococcus aureus*, *Enterococcus faecalis*, and *Streptococcus pneumoniae* have two copies of MurA, where the second is often called MurZ, MurA2 or MurAB (Blake et al., 2009; Mascari et al., 2022). These proteins are involved in normal growth, making them ideal drug targets. The inhibitor fosfomycin targets MurA in many species (Silver, 2017), however, a variation in a key amino acid residue in MurA homologs make this inhibitor ineffective in mycobacteria (De Smet et al., 1999).

As the first committed enzyme in an essential biosynthetic pathway, MurA is highly regulated. In *Listeria monocytogenes*, *Bacillus subtilis*, and *E. faecalis*, MurA activity is regulated through its degradation (Kock et al., 2004; Mascari et al., 2023; Wamp et al., 2020). In *L. monocytogenes*, ReoM interacts with MurA to promote its degradation by ClpCP, and this degradation is inhibited when ReoM is phosphorylated by PrkA (Wamp et al., 2020). A similar system involving kinase IreK and phospho-substrate IreB exists in *E. faecalis* (Mascari et al., 2023). Appropriate regulation of MurA stability is critical for survival and antibiotic resistance in *L. monocytogenes* and *E. faecalis* (Mascari et al., 2022; Wamp et al., 2022).

In mycobacteria, MurA’s enzymatic activity is directly activated by regulator CwlM, upon CwlM’s phosphorylation by PknB at its C-terminal tail (Boutte et al., 2016). CwlM is dephosphorylated during starvation (Boutte et al., 2016; Shamma et al., 2021), which presumably helps to downregulate MurA during growth arrest.

**FIGURE 1:**
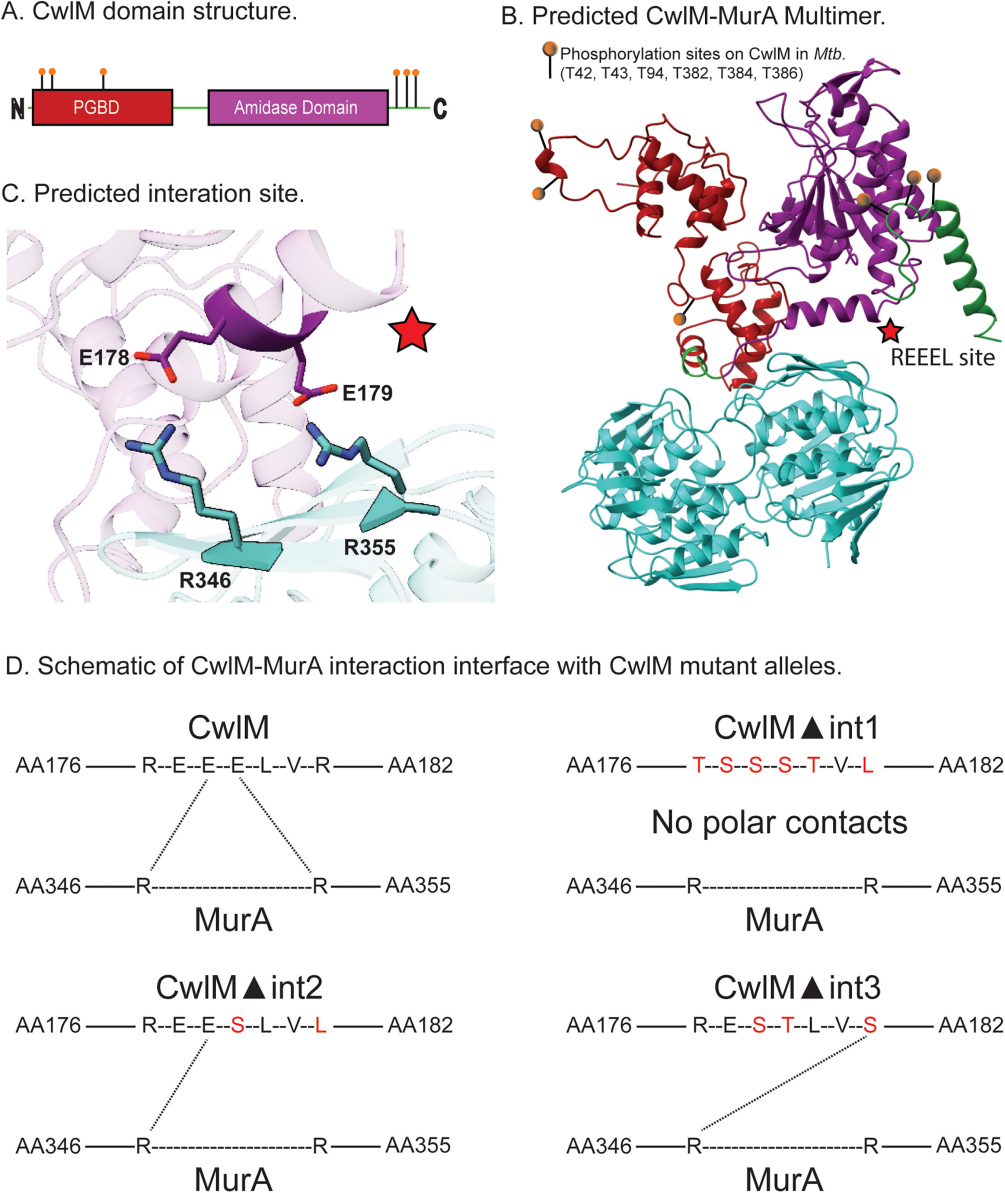
AlphaFold2 prediction of CwlM-MurA interaction complex and schematic of CwlM mutants with predicted polar contacts. (A) Domain structure of CwlM; the peptidoglycan binding domains (red), Amidase domain (purple), and phosphorylation sites (orange). (B) Multimer prediction of CwlM*_*Mtb*_* (top) and MurA*_*Mtb*_* (bottom), showing predicted interaction interface (red star; REEEL site), and phosphorylation sites (orange). (C) Close-up of the REEEL site, highlighting polar contacts between CwlM*_*Mtb*_* and MurA*_*Mtb*_*. Glutamate 178 and 179 on CwlM*_*Mtb*_* (purple), and arginine 346 and 355 on MurA*_*Mtb*_* (teal). (D) Schematic of CwlM and CwlM mutant alleles against MurA. Polar contacts were predicted from independent multimer predictions between each CwlM mutant (▲int1, ▲int2, and ▲int3) and MurA. For our purposes, ▲int represents the disruption of the predicted interaction between CwlM and MurA. The dotted lines represented Alphafold’s predicted polar contacts between residues of CwlM mutants and MurA. Amino acid changes are depicted in red.

In this work, we analyzed the effects of a predicted interface between CwlM and MurA on MurA activity *in vitro*, and on mycobacterial cell physiology. We identified a possible CwlM-MurA regulatory and interaction site using AlphaFold2 Multimer (Figure 1 and Supplementary Figure 1). We made mutants of CwlM that are predicted not to interact with MurA and show they are defective in activation of MurA *in vitro*. We tried several experiments to test the protein interaction between MurA and CwlM, but we were not able to measure this interaction, despite its clear regulatory role *in vitro*: this suggests that the interaction is transient and weak. During *in vivo* experiments in *Mycobacterium smegmatis* (*Msmeg*), we found that ablation of the predicted regulatory site prevented cell survival, while more conservative mutations caused a small decrease of peptidoglycan metabolism *in vivo.*

## Materials and methods

### AlphaFold predictions

AlphaFold monomer and multimer predictions (Jumper et al., 2021) were performed at the Texas Advanced Computing Center (TACC) at the University of Texas at Austin. Sequences were submitted remotely through Windows PowerShell and ran on TACC’s Lonestar6 High Performance Computer system.

### Media and culture conditions

All *E.coli* cultures were started with Luria-Bertani (LB) medium and shaken overnight at 37 °C. All *MSmeg* mc^2^155 wild-type and mutant cultures were started in 7H9 (Becton, Dickinson, Franklin Lakes, NJ) medium with 5 g/liter bovine serum albumin, 2 g/liter dextrose, 0.85 g/liter NaCl, 0.003 g/liter catalase, 0.2% glycerol, and 0.05% Tween 80 and shaken overnight at 37 °C until cultures entered logarithmic phase. For starvation and other specific assays, Phosphate buffer saline (PBS) and Hartmans-de Bont (HdB) minimal medium was made as described previously (Hartmans et al., 2006; Shamma et al., 2022). Cultures were inoculated to OD_600_ 0.05, unless otherwise stated. All CFU time points were plated on LB agar, and placed in 37 °C incubator for 3–4 days.

### Protein expression and purification

All proteins were expressed using *E.coli* BL21 Codon Plus, and all proteins were expressed from pET28 (MurA) or pET-his-SUMO (CwlM) derived expression vectors. Starter cultures were inoculated in 150 mL of LB medium, then incubated overnight at 37 °C with the appropriate antibiotic. The following day 1 L LB cultures were inoculated to an OD of 0.2, and shaken at 37 °C until OD 0.8. Cultures were then placed on ice for 30 min prior to induction. N-terminal tagged His-MurA_*Mtb*_ cultures were expressed for 4 h with 1 mM of isopropyl-B-D-thiogalactopyranoside (IPTG) at 16 °C. The 1 L cultures were then pelleted down at ∼1,800 g for 20 min at 4 °C. Pellets were then resuspended with cold MurA lysis/wash buffer (50 m Bis-Tris propane pH 7.5, 500 mM NaCl, 2 mM TCEP, 5 mM Imidazole, 10% glycerol). Resuspended cells were then lysed using the Emulsiflex C-5 instrument (AVESTIN). Lysates were then pelleted at 35,000 g for 35 min at 4 °C. Supernatants were filtered, then loaded on a Ni-NTA resin column (Econofit Nuvia IMAC #12009287). Nickel bound proteins were then washed and eluted with MurA buffer containing 5 and 500 mM imidazole, respectively. Elution fractions were concentrated, and soluble proteins were separated from aggregates through a Sephacryl S-200 resin (Cytiva) filled column (GE Healthcare XK 26/70) in MurA SEC buffer (MurA wash buffer with no imidazole. Soluble proteins were combined (Supplementary Figure 3), concentrated and stored in 20% glycerol in the −80 °C freezer. N-terminal tagged His-SUMO-CwlM*_*Mtb*_* and His-SUMO-CwlM*_*Mtb*_* mutants were harvested and purified similarly as His-MurA*_*Mtb*_.* In short, CwlM*_*Mtb*_* was induced for 18 h overnight at 4 °C with 1 mM IPTG. CwlM buffer consisted of 50 mM Tris pH 7.5, 350 mM NaCl, 1 mM DTT, 10% glycerol, and imidazole (10 mM for wash, 250 mM for elution, no imidazole for size-exclusion).

### MurA kinetic assays

Protocol was done as previously described (Boutte et al., 2016) with minor modifications. In short, purified His-SUMO-CwlM*_*Mtb*_* samples were first subjected to a protease reaction with His-Ulp1 to cleave the His-SUMO tag. His-Ulp1 was incubated with His-SUMO-CwlM at a 1:10 ratio and incubated at room temperature for at least 40 min in protease buffer (50 mM Tris pH 8, 350 mM NaCl, 2% Igepal, 10 mM DTT before use). CwlM was then purified away from His-SUMO and His-Ulp1 by running samples through a nickel column. All flowthrough samples were collected, concentrated, and analyzed by SDS gel to verify the tag-less CwlM (bands at ∼44 kDa). All tag-less CwlM strains (WT and mutants) were either left unphosphorylated or were phosphorylated through a kinase reaction (1:10 His-MPB-PknB to CwlM, 1 mM MnCl2, 0.5 mM ATP). Kinase reactions were performed at room temperature in 30 μL reactions in kinase buffer; 50 mM Tris pH 7.5, 150 mM NaCl, 1 mM DTT. For unphosphorylated CwlM, setup was the same, but ATP was not added. For the enzymatic assay, equimolar amounts of His-MurA were added to phosphorylated and non-phosphorylated samples of CwlM (3 μg of each), followed by the addition of substrates; 20 mM phospho-enol-pyruvate, 100 mM UDP-GlcNac. Samples were then filled to a total volume of 90 μL with reaction buffer (50 mM Tris pH 8.0, 2 mM KCl, 2 mM DTT) and incubated at 37 °C for 30 min. After incubation, the reaction was quenched by the addition of 90 μL of 0.4 M KOH. Samples were then spun/filtered using a microcon filter for 30 min, flowthrough for each sample was collected and analyzed through High-Performance Liquid Chromatography as previously described (Boutte et al., 2016).

### Co-immunoprecipitation assay

His-SUMO-CwlM*_*Mtb*_* was first subjected to a protease reaction with His-Ulp1 similarly as described above. Pure CwlM*_*Mtb*_* was then split into two samples, one that would be phosphorylated through a kinase reaction with His-MBP-PknB (Shamma et al., 2021), and a second that would be kept unphosphorylated. His-MurA*_*Mtb*_*-strep was expressed and purified the same way as His-MurA*_*Mtb*_*. Pure His-MurA*_*Mtb*_*-strep was then immobilized in two microtubes (3 μg in each) containing magnetic Strep-Tactin beads (IBA Lifesciences, Göttingen Germany) and washed with PBS buffer. Equimolar amounts of phosphorylated CwlM*_*Mtb*_* (3 μg: CwlM and MurA are both 40 kD) in PBS was added to one tube containing His-MurA*_*Mtb*_*-strep and beads, while equimolar unphosphorylated CwlM*_*Mtb*_* was added to the second tube. Tubes were incubated at room temperature for 3 min with some intermittent “flicking” to avoid settling of beads. Samples were placed in a magnetic rack and supernatant was collected and labeled as flow-through. Tubes were then taken off the magnetic rack and resuspended with PBS. Incubation and removal of supernatant was repeated one more time, this sample was labeled as wash. Next, beads were resuspended with 1x Laemmli buffer and cooked for 10 min. Tubes were then placed back on the magnetic rack and supernatant was collected. These samples were labeled as elution. Flowthrough, wash, and elution were then run on an SDS protein gel and analyzed via silver stain.

### CwlM allele survival

First, *cwlM*_*Msmeg*_ wild-type and mutant alleles were cloned into a kanamycin-marked L5 integrating vector (Hartmans et al., 2006). These vectors were then transformed into *Msmeg* strains with the native *cwlM* and *murA* knocked out, with nourseothricin-marked wild-type *cwlM* at the L5 integrating site, and either wild-type *murA* (CB737) or suppressor *murA* (CB762) at the tweety integrating site. These transformants were then plated on LB agar containing kanamycin, and then patched on nourseothricin and kanamycin after the colonies grew. Proper allele swapping and survival was assessed by counting the fraction of colonies that were kanR and nuoS, over the total number of kanR colonies. The allele survival was evaluated for 150–200 colonies for each *cwlM* allele.

### Growth curves of *cwlM* alleles

Biological triplicate cultures were grown for each *cwlM* allele in both *murA* backgrounds (CB737 and CB762) until log-phase. Growth curves were done on a non-tissue culture treated 96-well plate in 200 μL of 7H9 with a starting OD of 0.1 for each culture. The OD_600_ of each culture was measured every 30 min in a BioTek Synergy Neo2 muti-mode plate reader (Agilent, Santa Clara, CA). Data and doubling time were analyzed using GraphPad Prism software. Doubling time was calculated with a non-linear curve fit method using an exponential growth equation with the least squares fit. *P*-values were calculated using two-tailed-unpaired *t*-test.

### Cell staining and microscopy

Triplicate cultures were grown into log-phase in HdB minimal medium. After normalizing to OD 0.3, 1 mL of each replicate culture was stained with 10 μM HADA (R&D systems) for 15 min with rolling incubation at 37 °C. Post incubation samples were pelleted and resuspended once in 100–200 μL of HdB medium. Cells were then analyzed with a Nikon Ti-2 widefield microscope. A 350/50 nm excitation filter, 460/50 nm emission filter, and a 400 nm dichroic mirror was used to detect HADA signal. The image analysis for each sample was done using the MicrobeJ plugin through the ImageJ (FIJI) computer software. The mean intensity of about 300 cells per sample was then analyzed using GraphPad prism software. p-values were calculate using the student’s *t*-test.

### Cloning and plasmid construction

All other plasmids were made by cloning genes in previously-described plasmids using restriction enzymes to digest the vectors, and Gibson cloning (Gibson et al., 2009) to insert the described genes. The Δint1-3 point mutants of *cwlM* were made by PCR stitching. All the plasmids and strains built are listed in Supplementary Table 2. The primers used for cloning are listed in the Supplementary Table 3 and cross-referenced with the plasmid and strain list.

All pUAB plasmids were provided by Dr. Adrie Steyn (University of Alabama, Birmingham). In short, pUAB plasmids were digested to remove its GCN4 dimerization domain and replace it with bait or prey protein to generate fusion proteins with dihydrofolate reductase domains [either mDHFRF (F1,2) or mDHFRF (F3)]. pUAB100 was digested with BamHI-HF and ClaI. pUAB300 was digested with BamHI-HF and HpaI. pUAB200 was digested with MfeI and ClaI, while pUAB400 was digested with MfeI and HpaI. WT *cwlM*_*Mtb*_ and WT *murJ*_*Mtb*_ intracellular domain (residues A548 to N978) were cloned into each plasmid using Gibson cloning (Gibson et al., 2009). All plasmids were transformed into chemically competent *E.coli* Top10 cells and selected post-transformation in kanamycin and hygromycin containing LB Lennox plates. Post-sequence verification, each plasmid was transformed with its respective counterpart into electrically competent *Msmeg mc^2^155* cells.

### Mycobacterial protein fragment complementation assay

pUAB-containing experimental strains, including negative and positive controls, were grown to stationary phase in 7H9 (BD, Sparks, MD) liquid medium, and supplemented with 0.2% glycerol, 0.05% Tween80, and ADC (5 g/L albumin, 2 g/L dextrose, 0.85 g/L NaCl, and 0.003 g/L catalase). Cultures were then back diluted to maintain cells in logarithmic growth phase for 24 h. A total of 10 μL of log-phase culture for each strain was spotted onto 7H11 (Middlebrook) agar with 0.5% glycerol, 0.5% glucose and 0.2% Tween80, and supplemented with kanamycin (50 μg/mL), hygromycin (50 μg/mL) and/or trimethoprim (15 μg/mL) when necessary. Images were taken using a FOSITON Photo Light Box.

## Results

### AlphaFold2 predicts a charged interface between CwlM and MurA

Phosphorylated CwlM (CwlM∼P) activates MurA *in vitro*, and CwlM and MurA co-immunoprecipitate after cross-linking in *Msmeg* lysates (Boutte et al., 2016), suggesting that the two proteins interact. To identify a possible interaction site, we ran an AlphaFold2 Multimer (Jumper et al., 2021) prediction of the CwlM*_*Mtb*_* - MurA*_*Mtb*_* hetero-dimer (Figures 1B, C and Supplementary Figure 1). The prediction reveals two salt bridges (Figure 1C) between an alpha-helix on CwlM*_*Mtb*_* - which we named “REEEL” due to the amino acid sequence - and a beta-sheet on MurA*_*Mtb*_*. Glutamates 178 and 179 from CwlM*_*Mtb*_* are predicted to form polar contacts with arginines 346 and 355 of MurA*_*Mtb*_* (Figure 1C). Additionally, we identified two hydrogen-bond interactions upstream of the REEEL site: aspartate 348 on MurA with the backbone of Serine 171 on CwlM, and histidine 350 on MurA with the backbone of aspartate 117 on CwlM. The structural prediction at the interface is supported by confident pLDDT scores (Supplementary Figure 1) and consistent pTM and ipTM values (Supplementary Table 1.), indicating reliable overall folding and subunit positioning within the complex.

### Predicted salt bridges between CwlM and MurA are vital for MurA enzymatic activity *in vitro*

To test how the predicted interface between CwlM*_*Mtb*_* and MurA*_*Mtb*_* impacts activation of MurA, we expressed and purified three variants of CwlM: CwlM ▲int1, ▲int2, and ▲int3, in which different combinations of residues in the REEEL region were mutated to disrupt the charged interactions (Figure 1D). All proteins were purified by nickel affinity and size-exclusion chromatography, and all eluted from the size exclusion column at the same time as the wild-type protein. We mutated most of the residues to serine or threonine to break the predicted salt bridges while keeping the surface residues polar to maintain solubility of the protein.

**FIGURE 2:**
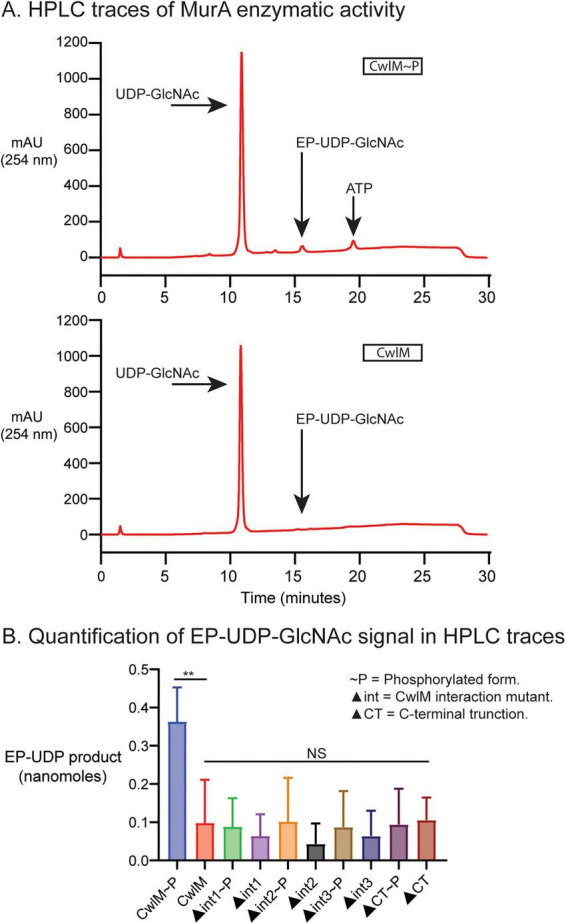
HPLC traces and product quantification of MurA enzymatic activity. (A) HPLC traces of MurA*_*Mtb*_* enzymatic assay in the presence of CwlM*_*Mtb*_*∼P (top) and CwlM*_*Mtb*_* (bottom). Arrows indicate the signal for the MurA substrate UDP-GlcNAc, the enzymatic product EP-UDP-GlcNAc, and ATP (CwlM∼P only). ATP is carry over from the phosphorylation reaction between CwlM and the kinase PknB. (B) Quantified signal of EP-UDP-GlcNAc for both phospho forms of CwlM, CwlM mutant alleles (▲int1, 2, 3), and CwlM with the C-terminal truncated (CT). Data are the mean of 3 or 4 replicate experiments with different preparations of MurA. There was no significant difference between CwlM, CwlM CT (∼P), and CwlM mutant alleles (∼P). The *P*-value between CwlM and CwlM∼P was 0.004. Asterisks represent significance as measured by the two-tailed Student’s *t*-test: **, *P ≤* 0.01.

Previous experiments show that unphosphorylated CwlM has poor activation of MurA’s enzymatic activity (Boutte et al., 2016). We measured MurA product formation *in vitro* through HPLC after incubation with substrates and wild-type or mutated CwlM proteins, in both phosphorylated and unphosphorylated states (Figure 2 and Supplementray Figure 2). We validated the peak that represented the enoyl-pyruvyl-UDP-GlcNAc product of MurA using MurA from *E. coli* (Supplementray Figure 2). Using wild-type CwlM*_*Mtb*_*, we confirmed the previous result that (Boutte et al., 2016) phosphorylation of CwlM stimulates MurA*_*Mtb*_* activity (Figure 2A). As a negative control, we tested a C-terminal truncation of CwlM*_*Mtb*_*, which has reduced activation of MurA, and found that, like unphosphorylated CwlM, it failed to substantially activate MurA (Figure 2B). MurA is also not activated by ATP alone (Supplementray Figure 2). Compared to wild-type CwlM∼P, all mutants were significantly impaired in MurA activation, regardless of phosphorylation (Figure 2B). The phosphorylated CwlM REEEL site mutants show the same deficit in MurA activation as unphosphorylated CwlM. We conclude that the REEEL site on CwlM is necessary for the activation of MurA*_*Mtb*_ in vitro*, and that phosphorylation alone is not enough to rescue activation. These results show that when CwlM and MurA are the only proteins present, CwlM requires both phosphorylation on its C-terminal tail and charged residues on the REEEL-alpha helix to activate MurA’s enzymatic activity.

### Interaction between CwlM and MurA could not be detected

Previous pulldown assays showed that FLAG-tagged CwlM could be co-immunoprecipitated with MurA-strep in cross-linked *Msmeg* lysates, suggesting an interaction between the two proteins (Boutte et al., 2016). We tested if we could observe this interaction with purified proteins, without cross-linking and in the presence of MurA substrates. We immobilized His-MurA*_*Mtb*_*-strep on anti-strep magnetic beads and poured over equimolar CwlM*_*Mtb*_* or CwlM*_*Mtb*_*∼P in buffer containing substrates. We found that CwlM and CwlM∼P interact with the beads, and we could not observe interactions with MurA that were above the background (Supplementray Figure 4). We found similar results when using nickel beads to immobilize His-MurA, and we were also unable to validate an interaction using biolayer interferometry (BLI) (data not shown). We further tested the interaction *in vivo*, using a two-hydrid protein fragmentation assay (Singh et al., 2006), but again observed no detectable interaction (Supplementray Figure 8). Lastly, we also tested the interaction *in vivo* using the split-GFP system (Cabantous et al., 2005), but again could not measure a significant interaction (data not shown). Our results suggest that the interaction between MurA and CwlM is transient or very weak under both *in vitro* and *in vivo* conditions.

### CwlM regulatory mutants exhibit minimal phenotypes in terms of growth and cell wall defects

We sought to determine how *cwlM* mutants with decreased activation of MurA *in vitro* affect cell physiology. To do this, we constructed strains of *Msmeg* carrying either wild-type or mutant alleles of *cwlM* in a wild-type *murA* or *murA* S368P genetic background. The MurA S368P regulatory mutant is hyperactive in the absence of CwlM regulation, so *cwlM* mutants that are specifically defective in MurA activation display reduced phenotypic defects in this genetic background (Boutte et al., 2016).

**FIGURE 3:**
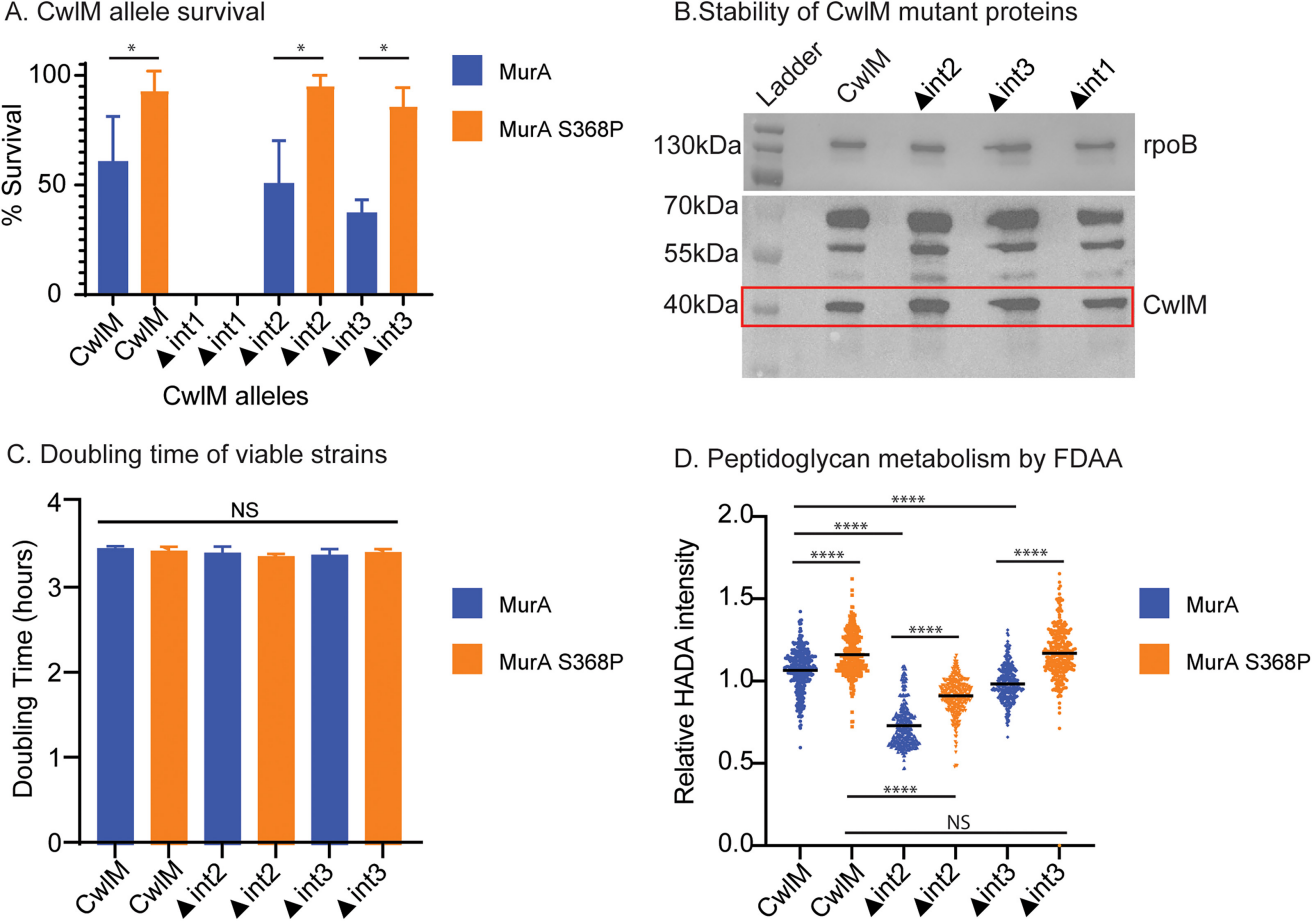
Effects of *cwlM* alleles on mycobacterial cell physiology. (A) Survival of *cwlM* alleles in wild-type *murA* (blue) and *murA* suppressor (S368P) (orange) backgrounds. Survival was assessed by the percent of colonies in which the wild-type *cwlM* allele was replaced by the indicated mutant allele (wild-type, ▲int1, ▲int2, ▲int3). Four biological replicates of each transformation were performed, and 40-50 colonies were counted for each replicate. There was no survival/recovery for ▲int1. (B) Anti-HA western blot of *cwlM*-HA and *cwlM*-HA mutant alleles (∼44 kDa) in a merodiploid background. CwlM runs at just above 40 kD, see Fig. S3. rpoB was used as an internal loading control (∼160 kDa). Spot intensities were analyzed with Fiji (imageJ) software, there was no significant difference between strains. (C) Doubling time of strains carrying *cwlM* alleles recovered from allele swaps (A) in both *murA* backgrounds. Doubling times were extracted from two growth curve experiments done in triplicates (Supplementary Figure 5). Prism software was used to perform multiple comparisons using One-way ANOVA, there was no significant difference between strains. (D) Relative mean HADA intensity per cell of *cwlM* alleles in both *murA*backgrounds. Asterisks represent significance as measured by the two-tailed Student’s *t*-test: *, *P ≤* 0.05; **, *P ≤* 0.01; ***, *P ≤* 0.001; ****, *P ≤* 0.0001; NS, *P >* 0.05.

We started first by assessing the efficiency with which different alleles of *cwlM* could be exchanged using the L5 phage integrase system (Pashley and Parish, 2003). We found that allele swapping was more efficient in the *murA S368P* background than in the wild-type *murA* background (Figure 3A). For ▲int1, containing six amino acid mutations in the REEEL site (Figure 1D), there was no recovery during allele swapping in either *murA* background (Figure 3A). This result indicates that abrogation of the charged residues at this site either completely prevents MurA activation or has a detrimental effect on other functions of CwlM besides the activation of MurA. The ▲*int2* and ▲*int3 cwlM* alleles, which carry conservative mutations in the predicted CwlM–MurA regulatory interface were able to replace the wild-type allele at rates similar to wild-type (Figure 3A). We performed western blots on merodiploid strains containing wild-type *cwlM* in the native site and the different HA-tagged *cwlM* mutants at the L5 integration site. Results showed that all the CwlM mutant proteins were stable (Figure 3B).

To test whether the *cwlM* mutants had any growth defects, we performed growth curves (Supplementary Figure 5). The strains expressing only *cwlM*▲int2 and ▲int3 had the same doubling time as wild-type *cwlM* (Figure 3C).

Since MurA is the first enzyme in peptidoglycan precursor synthesis, we hypothesized that strains carrying *cwlM* alleles defective in MurA activation could alter peptidoglycan metabolism. To test this, we constructed strains of *Msmeg* carrying either wild-type or mutant alleles of *cwlM* in the MurA S368P genetic background - which has over-active MurA in the absence of CwlM activation (Boutte et al., 2016). We grew these strains, carrying different *cwlM* and *murA* alleles, to logarithmic phase and stained them with HADA, a fluorescent D-amino acid that is incorporated into metabolically active peptidoglycan (Kuru et al., 2012). HADA is incorporated largely by LD-transpeptidases in mycobacteria: changes in HADA signal are due to a change in new cell wall synthesis and altered LDT activity (Baranowski et al., 2018; García-Heredia et al., 2018).

Results from HADA staining and microscopy analysis showed decreased HADA signal in strains with *cwlM*▲int2 and ▲int3 compared to *cwlM* wild-type in the wild-type *murA* background, with a larger defect in the *cwlM*▲int2 strain (Figure 3D). All strains with the *murA* S368P allele stained more brightly than the isogenic strains with the wild-type *murA*, regardless of the *cwlM* allele: this supports the model that the *cwlM* mutations could work through regulation of MurA, as an over-active mutant of MurA can override the defects of the *cwlM* mutations. The *cwlM*▲int2 strain had a significant defect compared to the *cwlM* WT in the *murA* S368P background (Figure 3D). Although we see a difference in HADA staining, there was no difference in cell length between *cwlM* alleles and *murA* backgrounds (Supplementary Figure 6A), and modest differences in mean cell width (Supplementary Figures 6B–D).

Taken all together, our results show that the mutants of CwlM that are defective in activation of MurA *in vitro* have very modest impacts on peptidoglycan metabolism *in vivo.* This result indicates that the regulation of MurA by CwlM that we can measure *in vitro* is only a small component of MurA regulation *in vivo.* Our result that the *cwlM* ▲int1 mutant strain was not viable (Figure 3A) suggests that this interaction may interfere with another function of CwlM. CwlM has been shown to interact with MurJ and FhaA (Turapov et al., 2018), though the regulatory significance of those interactions has not been described. Because MurJ is essential (DeJesus et al., 2017) and involved in peptidoglycan metabolism (Sham et al., 2014), we sought to further explore the interaction between CwlM and MurJ. The Mycobacterial two-hybrid assay (Singh et al., 2006) was previously used to confirm the interaction between CwlM and the MurJ cytoplasmic domain (Turapov et al., 2018). We re-built the described constructs; however, we could not confirm the interaction between CwlM and MurJ (Supplementary Figure 7).

## Discussion

MurA is an essential enzyme for peptidoglycan metabolism (Marquardt et al., 1992) and as the first enzyme in the peptidoglycan precursor pathway, its regulation affects the whole pathway. In other bacteria, MurA is regulated by proteolysis (Wamp et al., 2022), transcription (Blake et al., 2009), and protein-protein interactions (Hummels et al., 2023). MurA is also regulated in response to environmental factors encountered during infection (Fisher et al., 2002; Wu et al., 2012). Thus, regulation of MurA is important for infection and stress responses broadly.

Using AlphaFold2 we predicted a charged interface between regulator CwlM and MurA (Figure 1). We were able to demonstrate the importance of the predicted regulatory interface for MurA enzymatic activity using *in vitro* biochemistry (Figure 2 and Supplementary Figure 2), which highlights that activation of MurA requires both phosphorylation of CwlM and this charged interface, which is not near the phosphorylation site at the C-terminus. In our biochemical assays, we measured the accumulation of the MurA product, enoyl-pyruvyl- UDP-GlcNAc (Marquardt et al., 1992) by HPLC. Often MurA activity has been studied by measuring the accumulation of a byproduct of the reaction (Marquardt et al., 1992; Xu et al., 2014) – a malachite green assay is used to measure P_*i*_ that is released from the phospho-enol-pyruvate precursor (Lanzetta et al., 1979). We were unable to use this assay reliably – we believe this is because the ATP added for phosphorylation of CwlM caused increases in P_*i*_ levels that masked the signal. While measuring products is a more direct way to study enzymes than measuring byproducts, a downside of this approach is the difficulty in comparing MurA activity in our assays to the activity measured in the assays of other MurA proteins studied by other groups. We did validate our assay by showing that MurA from *E. coli* produced a product that eluted from the HPLC column at the same time as the product from MurA*_*Mtb*_* (Supplementary Figure 2B). Another shortcoming of our study is that we did not make mutants of MurA that would re-constitute the CwlM-MurA salt bridge interactions at the REEEL site. This means that we cannot conclude that the formation of the salt bridges specifically is required for activation, just that the residues on CwlM that we mutated are required for MurA activation.

Despite trying many different assays including *in vitro* co-immunoprecipitation (Supplementary Figure 4) and Mycobacterial two-hybrid (Supplementary Figure 8) as well as Biolayer interferometry and split-GFP association assays (data not shown), we were not able to measure an interaction between CwlM or CwlM∼P and MurA. We acknowledge that there are protein interaction assays that we did not test, such as *in vivo* immunoprecipitations with crosslinking. This suggests that the interaction between CwlM(∼P) and MurA is weak, and that affinity between these proteins may not be regulated during MurA activation *in vitro*. Our data support a model in which phosphorylation of CwlM and the presence of charged residues in the REEEL region are involved in functional activation; however, we remain uncertain about how these changes affect the weak affinity between these two proteins.

Our *in vivo* results confirm that the charged CwlM-MurA interface affects peptidoglycan metabolism (Figure 3), but suggests that this regulation by CwlM has only a small role in regulating MurA activity in the cell (Figure 3D). We expect that additional regulatory systems help control MurA activity in the cell, as the *in vitro* activity we measured— even in the presence of CwlM∼P and high substrate concentrations— remains relatively low (Figure 2; Boutte et al., 2016). Moreover, the Km values we previously measured are significantly higher than the expected intracellular concentrations of these metabolites (Boutte et al., 2016). Additionally, the modest *in vivo* effects that we observed could be due to other regulatory systems circumventing the defects in MurA activity, such as upregulation of peptidoglycan recycling, which could compensate for decreased synthesis of enoylpyruvate-UDP-GlcNAc, the MurA product (Borisova et al., 2016).

Previous work showed that CwlM is essential even in a background expressing MurA S368P, which is more active in the absence of CwlM (Boutte et al., 2016), suggesting that CwlM has other essential roles in addition to activating MurA (Turapov et al., 2018). Overall, our findings indicate that mycobacterial MurA is regulated through at least two sites with CwlM. Further studies describing the regulatory systems governing MurA activity and its impact on cell wall biosynthesis may offer new avenues for the development of antimicrobial strategies for *Mtb*.

## Data Availability

The raw data supporting the conclusions of this article will be made available by the authors, without undue reservation.
