# Supplementary material for: The in vitro stimulatory activity of CwlM∼P on mycobacterial MurA depends on a charged predicted interface that is distal from the CwlM phosphorylation site
Source: Front Microbiol. 2025 Dec 9;16:1543775. doi: 10.3389/fmicb.2025.1543775 (PMC12723410; doi:10.3389/fmicb.2025.1543775)
Supplement: Supplementary file 1 [file Data_Sheet_1.PDF]

Supplemental Material

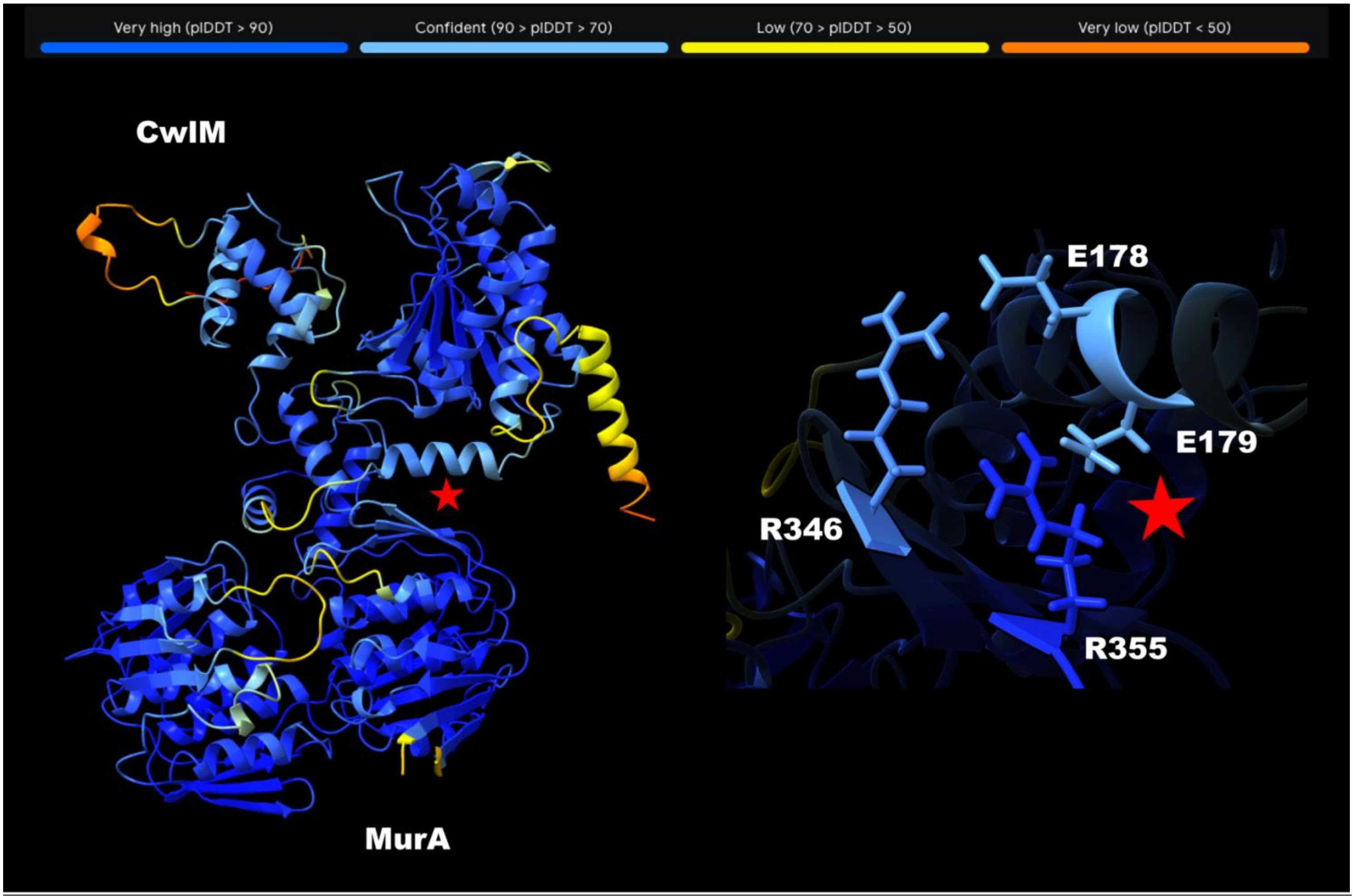

Supplemental Figure 1. Confidence analysis of CwlM*Mtb* and MurA*Mtb* predicted interaction using AlphaFold3 (1). pLDDT values were extracted and analyzed from AlphaFold3 prediction using ChimeraX software. Highlighted here, glutamate 178 and 179 on CwlM, and arginine 346 and 355 on MurA. Model confidence colors are as follows: blue, very high confidence (>90); cyan/light blue, confident (90>70); yellow, low confidence (70>50); orange, very low confidence (<50). Our original structure prediction was performed on Alphafold 2 (Fig. 1). We re-ran the prediction on Alphafold 3 and got essentially identical results.

Supplemental Figure 2. HPLC traces of MurA enzymatic reaction in *Mtb* and *E.coli*.

A) HPLC trace of $\text{MurA}_{\textit{E.coli}}$ enzymatic reaction with MurA substrates in the abscence of ATP. Black: experimental; Red: control (No MurA). **B)** HPLC trace of $\text{MurA}_{\textit{Mtb}}$ enzymatic reaction with CwlM~P (black) and CwlM (Red). Product signal of EP-UDP-GlcNAc for all reactions is at a retention time of 15 min and 50 sec. **C)** Quantification of the EP-UDP-GlcNAc product for the ATP control compared to the No CwlM control, and the experimental runs of CwlM~P vs. CwlM. Asterisks represent significance as measured by the two-tailed Student's t test: *, $P \leq 0.05$; **, $P \leq 0.01$; ***, $P \leq 0.001$; NS, $P > 0.05$.

A. HPLC traces of MurA$_{E.coli}$ enzymatic reaction.

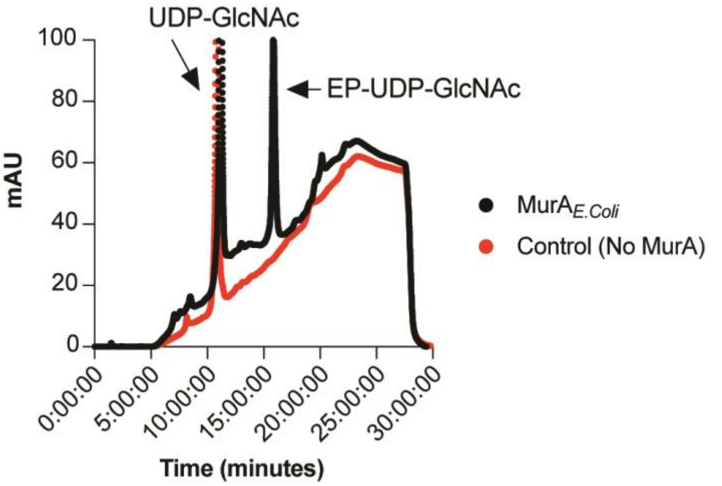

B. HPLC traces of MurA$_{Mtb}$ enzymatic reaction.

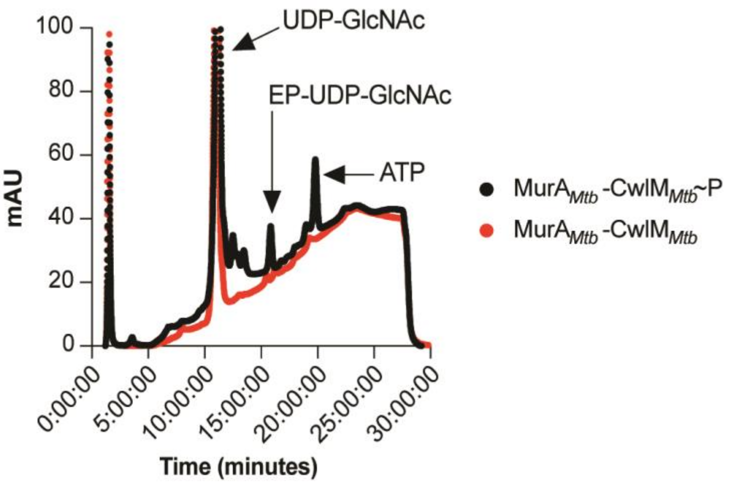

C. Quantification of MurA$_{Mtb}$ enzymatic reaction.

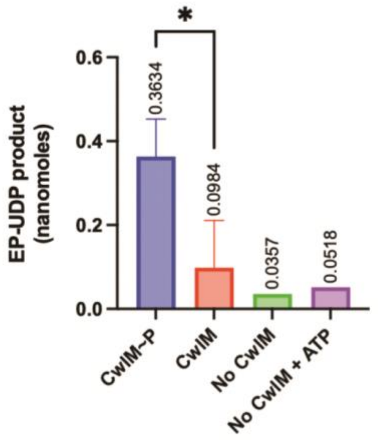

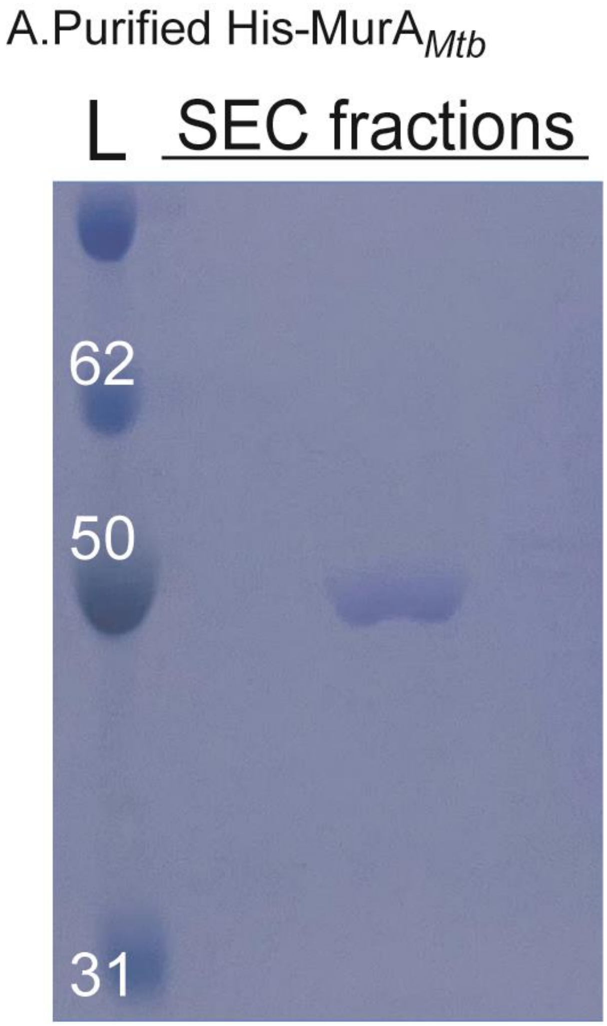

Supplemental Figure 3. SDS-PAGE analysis of His-MurA$_{Mtb}$ purification. A) SDS-PAGE of size-exclusion chromatography fractions corresponding to the elution peak at the expected molecular weight of His-MurA$_{Mtb}$. Fractions were selected based on the elution profile and compared to a standard linear calibration curve. Three fractions were analyzed; the middle lane contains the purified target protein. (L) Pre-stained protein standard (GenScript, M00624-250).

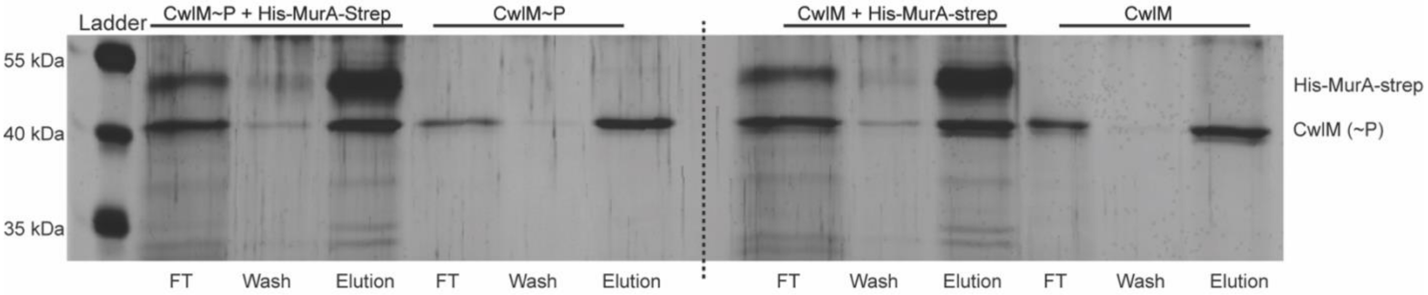

Supplemental Figure 4. CwlM and MurA do not interact in a co-immunoprecipitation assay in the presence of MurA substrates. Silver-stained protein gel of CwlM-MurA CO-IP using strep beads. Equimolar $CwlM_{Mtb}$(~P) and $MurA_{Mtb}$-strep were incubated with magnetic strep beads and MurA substrates. Control sample contained only CwlM(~P). Flowthrough (FT), wash, and elution samples were run for both phospho-forms, CwlM~P and CwlM on the left and right of the dotted line respectively. Spot intensities between experimental CwlM(~P) and control were analyzed using Fiji (ImageJ) software, there was no significant difference between control and experimental, and between both phospho-forms. The experiment was repeated with no substrates, with similar results (data not shown).

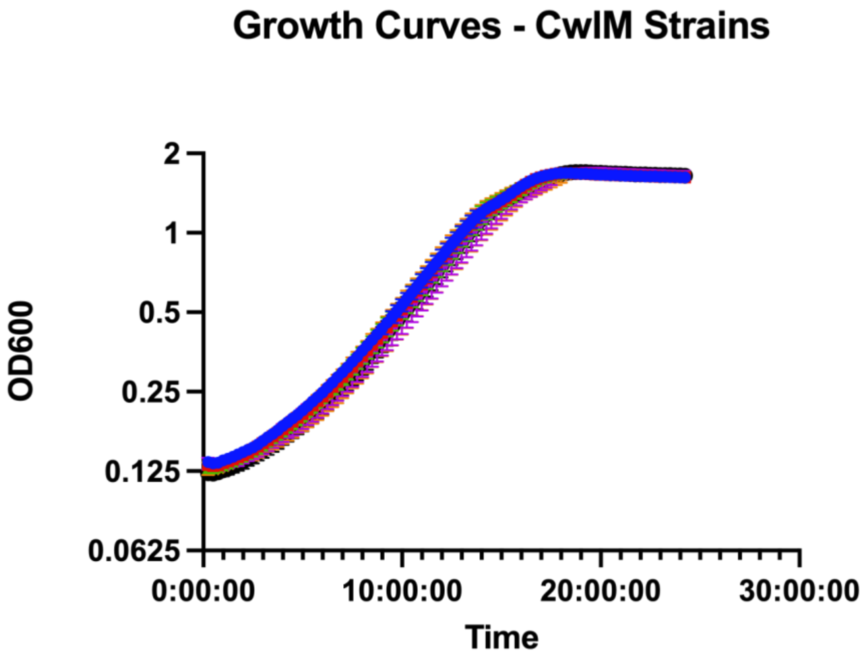

Supplemental Figure 5. Growth curves of *cwlM* alleles. Growth curves were performed in a 96-well plate using the Biotek Synergy Neo2 multi-mode plate reader. All the *cwlM* alleles in both wild-type MurA and MurA suppressor (S368P) background were grown in 7H9 medium for 24 h. Data was analyzed using GraphPad Prism software. There was no difference in growth between all the strains.

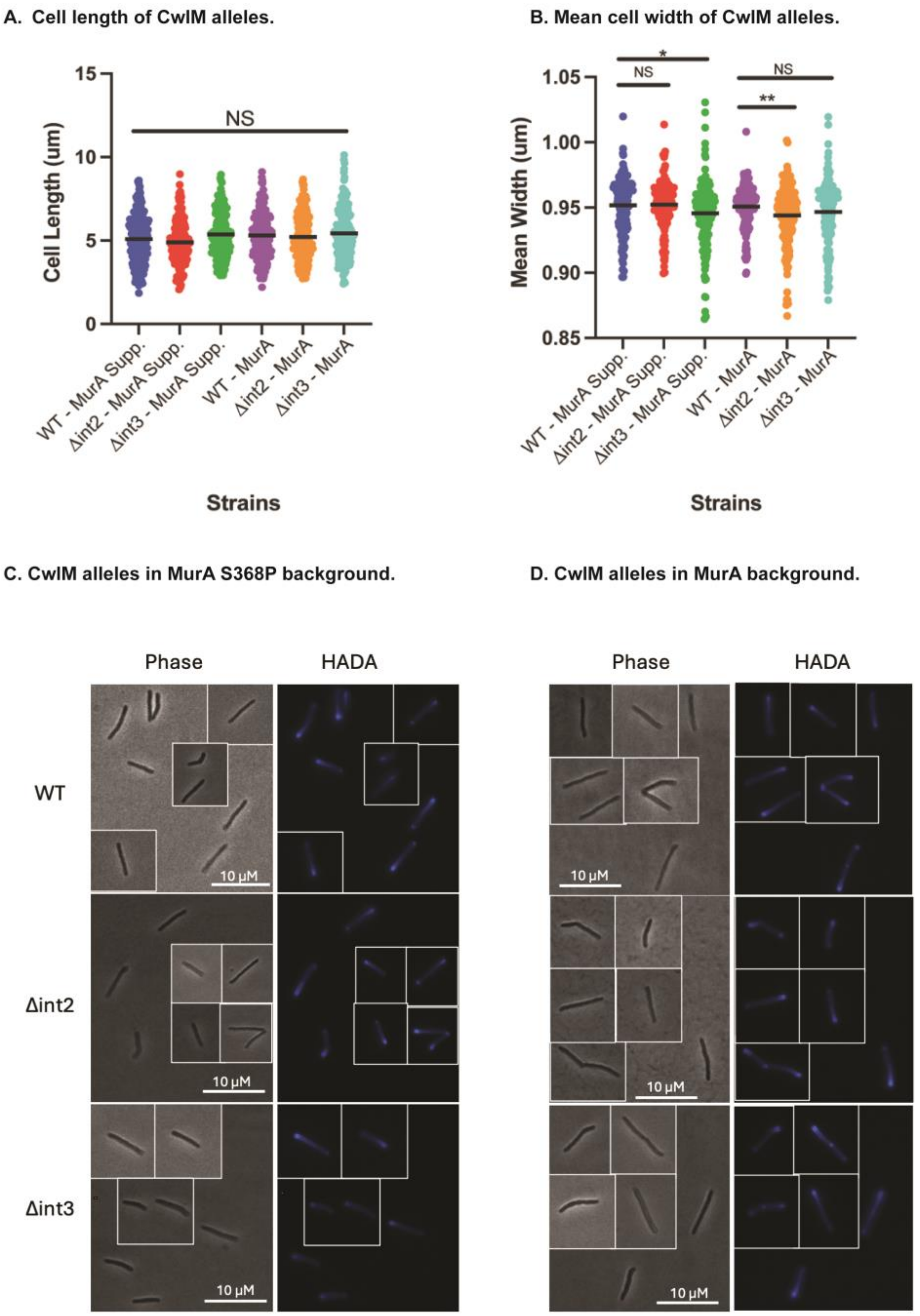

Supplemental Figure 6. Microscopy of *cwlM* allele strains. Cell length **(A)** and mean cell width **(B)** of *cwlM* alleles in both wild-type murA and *murA* suppressor background. Asterisks represent significance as measured by the two-tailed Student's *t* test: *, $P \leq 0.05$; **, $P \leq 0.01$; ***, $P \leq 0.001$; NS, $P > 0.05$. Microscopy images of *cwlM* alleles in MurA S368P suppressor background **(C)** and WT MurA background **(D).**

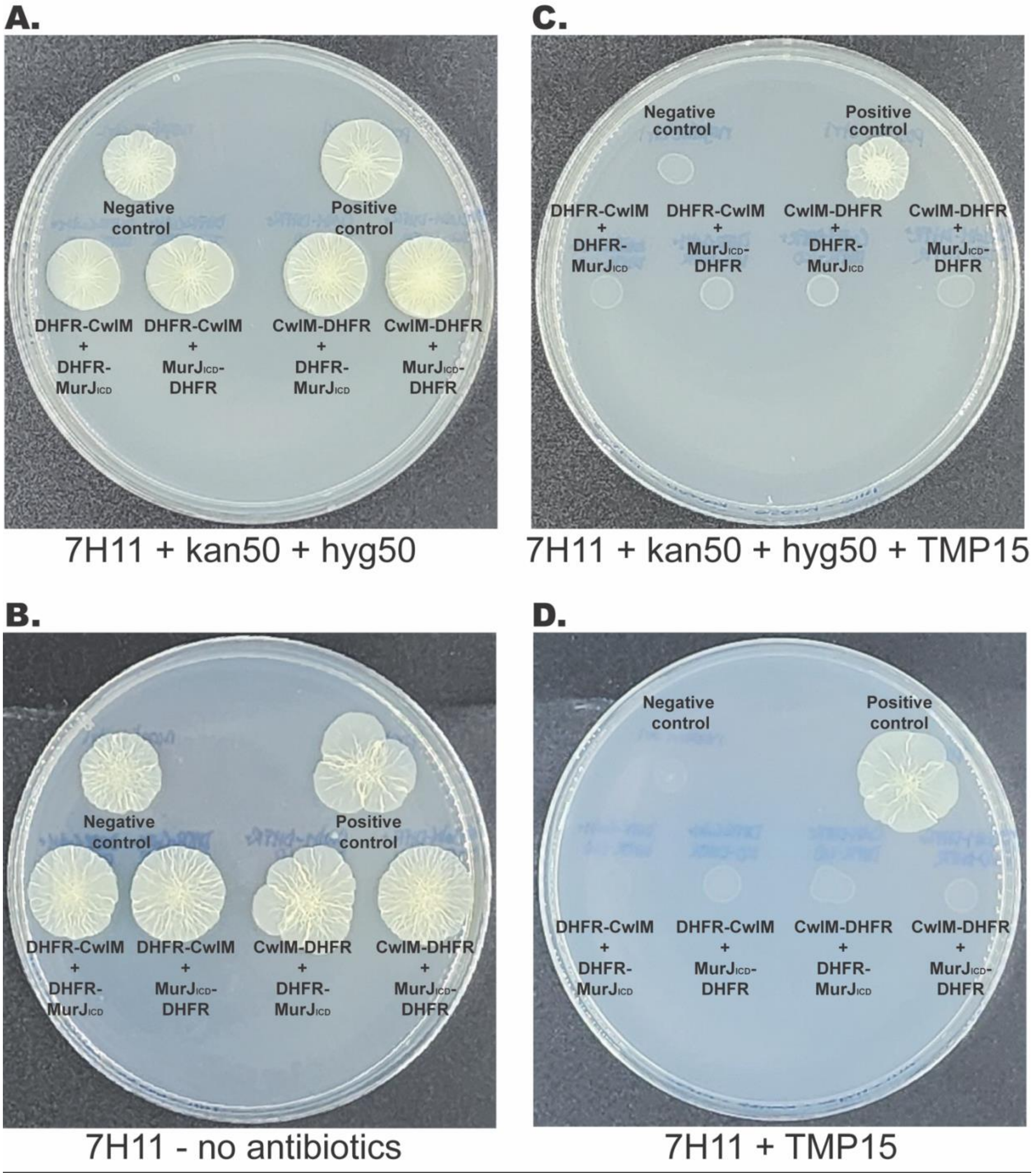

Supplemental Figure 7. CwlM$_{Mtb}$ does not interact with intracellular domain of MurJ$_{Mtb}$ (MurJ$_{ICD}$) by mycobacterial protein fragmentation assay (2). The negative control contains pUAB300-hsp60-DHFR[1,2] + pUAB400-hsp60-DHFR[3], and the positive control contains pUAB100-hsp60-GCN4 dimerization domain + pUAB200-

hsp60-GCN4-DHFR[3]. Growth in the presence of trimethoprim (TMP) indicates the reconstitution of dihydrofolate reductase (DHFR), indicating an interaction between the bait and prey proteins. The fragmented DHFR domains were cloned on either the N-terminal or C-terminal end of the bait or prey proteins. The position and orientation of the DHFR fragments are indicated on the labels; N-terminal on the left side (example: DHFR-CwlM), C-terminal on the right side (example: CwlM-DHFR). (A-B) Control plates +/- antibiotics, respectively. (C-D) Experimental plates to test interaction of CwlM and $MurJ_{ICD}$.

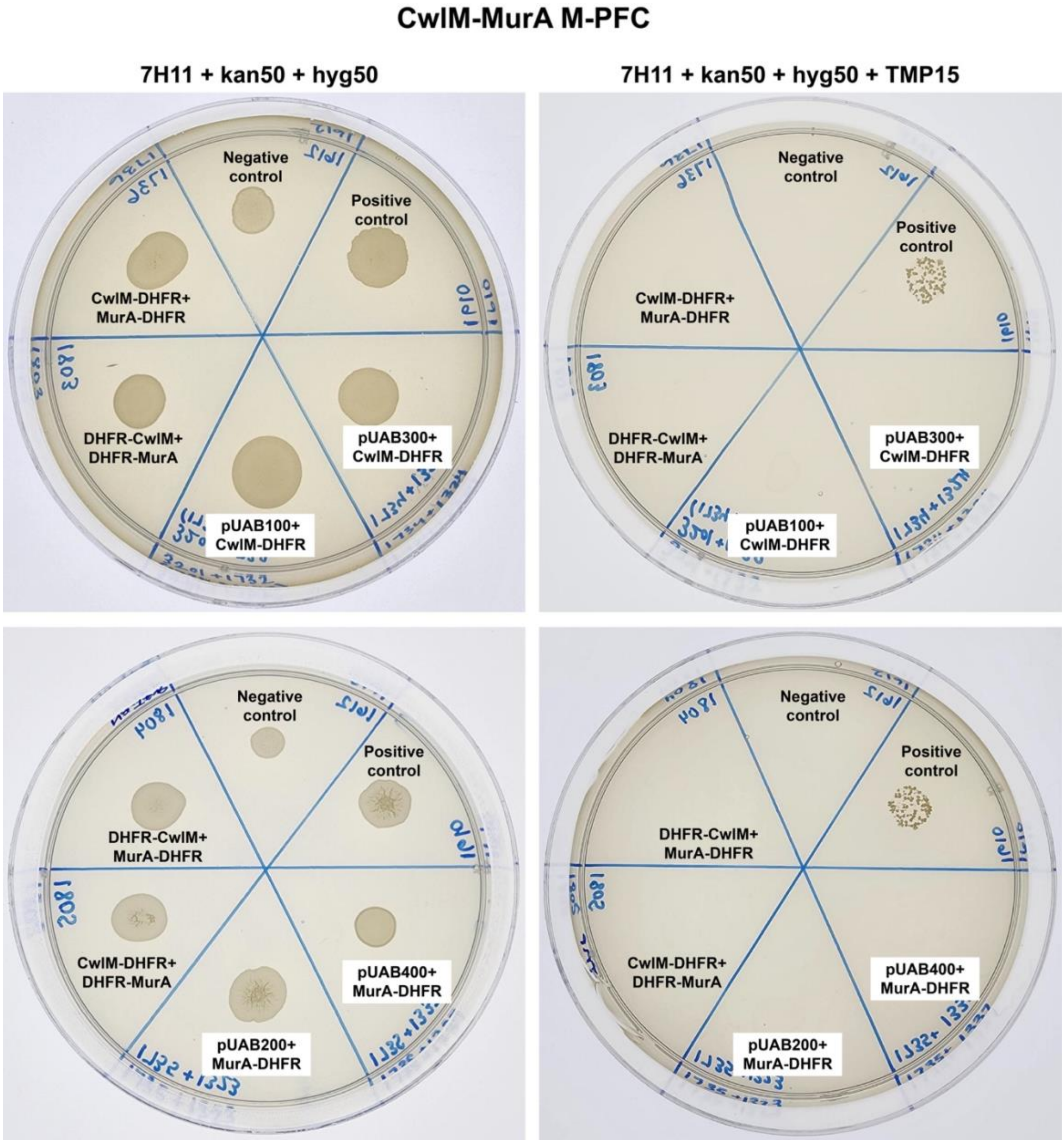

Supplemental Figure 8. Wild-type *Mtb* CwlM does not interact with WT *Mtb* MurA by mycobacterial protein fragmentation assay. The negative control is a strain containing pUAB300 + pUAB400. The positive control is a strain containing pUAB100 + pUAB200. Growth in the presence of trimethoprim indicates interaction between the bait and prey proteins which are fused to the fragmented dihydrofolate reductase domains. The positions and orientations of strains used are indicated in the pictures (and as previously mentioned in Fig. S9).

Supplemental Table 1. Predicted template modeling (pTM) and interface predicted template modeling (ipTM) scores for CwlM–MurA complex models.

**Multimer prediction**	**ipTM**	**pTM**
CwlM$_{Mtb}$-MurA$_{Mtb}$	0.76	0.72
CwlM$_{Mtb}$-Δint1--MurA$_{Mtb}$	0.76	0.68
CwlM$_{Mtb}$-Δint2--MurA$_{Mtb}$	0.75	0.7
CwlM$_{Mtb}$-Δint3--MurA$_{Mtb}$	0.75	0.7

AlphaFold-Multimer was used to generate structural predictions for CwlM–MurA complexes and their mutants. The **pTM** score reflects the overall confidence in the predicted fold of the complex, while the **ipTM** score estimates the confidence in the relative positioning of the subunits at the interface. Values above 0.8 indicate highly confident predictions; values between 0.6–0.8 fall within a moderate-confidence range; and values below 0.6 suggest unreliable or failed interface predictions.

Supplemental Table 2. Plasmids and strains.

**Strain #**	**nickname**	**genotype**	**Figure panel**	**Plasmid Citation (this paper if not indicated)**
CB174	pCT94	Top10 / pL5 PTetO Msm PonA1 truncation A-FLAG	3	(3)
CB1109	pET-his-SUMO	DH5a / pET-His-SUMO	2	(4)
CB1155	pET-his-SUMO-CwlM	Top10 / pET-His-SUMO-CwlM	2	
CB2796	pET-his-SUMO-CwlM (Δint1)	Top10 / pET-his-SUMO-CwlM TS (Δint1)	2	
CB3166	pET-his-SUMO-CwlM (Δint2)	Top10 / pET-his-SUMO-CwlM 3AA (Δint2)	2	
CB3167	pET-his-SUMO-CwlM (Δint3)	Top10 / pET-his-SUMO-CwlM V2 (Δint3)	2	
CB2736	pET-his-SUMO-CwlM (ΔCT)	Top10 / pET-his-SUMO-CwlM C-terminal trunc. (ΔCT)	2	
CB745	pET28 with N or C- terminal His-tag	DH5a / pET28	2	
CB2669	pET28-his-MurA	Top10 / pET28 – his-MurA(Mtb)	2	
CB1069	His-MPB-PknB (kinase domain)	DH5a / PHMGWA-PknB(KD)	2	(5)
CB1108	Ulp1-His	DH5a / pET-Ulp1	2	(4)
CB737	Parent strain for CB3042, CB3045, CB3048	mc^{2}155 ΔCwlM::loxP L5::KK158 - PCwlM-CwlM tw::pDE43MCtZ - PMurA-MurA-strep ΔMurA1::lox ΔMurA2::lox	3A, 3C, 3D	(6)
CB2835	pCB2835 -WT CwlM-Strep-HA	Top10 / pCT94-Msmeg WT CwlM-strep HA	3	
CB2911	pCB2911 -CwlM Δint1-Strep-HA	Top10 / pCT94-Msmeg CwlM-TS-strep-HA	3	

CB2995	pCB2995 -CwlM ∆int2-Strep-HA	Top10 / pCT94-Msmeg CwlM-3AA-strep-HA	3	
CB2996	pCB2996 -CwlM ∆int3-Strep-HA	Top10 / pCT94-Msmeg CwlM-V2-strep-HA	3	
CB3042	mc^{2}155 L5:pCB2835 Tw:MurA-strep	mc^{2}155 ∆CwlM::loxP L5::CwlM tw::pDE43MCtZ - PMurA-MurA-strep ∆MurA1::lox ∆MurA2::lox	3A, 3C, 3D	
CB3045	mc^{2}155 L5:pCB2995 Tw:MurA-strep	mc^{2}155 ∆CwlM::loxP L5::CwlM 3AA (CB2995) tw::pDE43MCtZ - PMurA-MurA-strep ∆MurA1::lox ∆MurA2::lox	3A, 3C, 3D	
CB3048	mc^{2}155 L5:pCB2996 Tw:MurA-strep	mc^{2}155 ∆CwlM::loxP L5::KK158 - CwlM V2 tw::pDE43MCtZ - PMurA-MurA-strep ∆MurA1::lox ∆MurA2::lox	3A, 3C, 3D	
CB3051	mc^{2}155 L5 :: pCB2835	mc2155 :: L5 :: pCT94-Msmeg WT CwlM-strep HA	3B	
CB2912	mc^{2}155 L5 :: pCB2911	mc2155 :: L5 :: pCT94-Msmeg CwlM-TS-strep-HA	3B	
CB3052	mc^{2}155 L5 :: pCB2995	mc2155 :: L5 :: pCT94-Msmeg CwlM-3AA-strep-HA	3B	
CB3053	mc^{2}155 L5 :: pCB2996	mc2155 :: L5 :: pCT94-Msmeg CwlM-V2-strep-HA	3B	
CB762	Parent strain for CB3033, CB3036, CB3039	mc2155 ∆CwlM::loxP L5::KK158 - PCwlM-CwlM tw::pDE43MCtZ - PMurA-MurA S368P-strep ∆MurA1::lox ∆MurA2::lox 1	3D	(6)
CB3033	mc^{2}155 L5::pCB2835 Tw::MurA S368P-strep	mc2155 ∆CwlM::loxP L5::CwlM (CB2835) tw::pDE43MCtZ - PMurA-MurA S368P-strep ∆MurA1::lox ∆MurA2::lox 1	3D	
CB3036	mc^{2}155 L5::pCB2995 Tw::MurA S368P -strep	mc2155 ∆CwlM::loxP L5::CwlM 3AA (CB2995) tw::pDE43MCtZ - PMurA-MurA S368P-strep ∆MurA1::lox ∆MurA2::lox 1	3D	
CB3039	mc^{2}155 L5::pCB2996 Tw::MurA S368P -strep	mc2155 ∆CwlM::loxP L5::CwlM V2 (CB2996) tw::pDE43MCtZ - PMurA-MurA S368P-strep ∆MurA1::lox ∆MurA2::lox 1	3D	
CB1338		pUAB100-HSp60-GCN4 dimerization domain-DHFR (1,2)	S 6	(2)
CB1323		pUAB200-hsp60-GCN4-DHFR (3)	S 6	(2)
CB1324		pUAB300-hsp60-DHFR (1,2)	S 6	(2)
CB1337		pUAB400-hsp60-DHFR (3)	S 6	(2)
CB1613	Positive control for M-PFC	mc^{2}155 L5::pUAB200-hsp60-GCN4-DHFR (3) / pUAB100-hsp60-GCN4-DHFR (1,2)	S 6	
CB1612	Negative control for M-PFC	mc^{2}155 L5::pUAB400-hsp60-DHFR (3) / pUAB300-hsp60-DHFR (1,2)	S 6	
CB3281	DHFR-CwlM + DHFR-MurJ$_{ICD}$	mc^{2}155 L5::pUAB400-hsp60-WT Tb MurJ$_{ICD}$-DHFR (3) / pUAB300-hsp60-DHFR (1,2)-WT Tb CwlM	S 6	
CB3277	DHFR-CwlM + MurJ$_{ICD}$-DHFR	mc^{2}155 L5::pUAB200-hsp60-WT Tb MurJ$_{ICD}$-DHFR (3) / pUAB300-hsp60-DHFR (1,2)-WT Tb CwlM	S 6	
CB3205	CwlM-DHFR + DHFR-MurJ$_{ICD}$	mc^{2}155 L5::pUAB400-hsp60-DHFR (3)-WT Tb MurJ$_{ICD}$ / pUAB100-hsp60-WT Tb CwlM-DHFR (1,2)	S 6	
CB3107	CwlM-DHFR + MurJ$_{ICD}$-DHFR	mc^{2}155 L5::pUAB200-hsp60-WT Tb MurJ$_{ICD}$-DHFR (3) / pUAB100-hsp60-WT Tb CwlM-DHFR (1,2)	S 6	

Supplemental Table 3. Primer list.

Strain #	Plasmid name	primers	Figure panel
CB1109	pET-His-SUMO	F1 – ggaattgtgagcggataacaattcc<br>R1 - gttatgctagttattgctcagcgg	2
CB1155	pET-His-SUMO-cwlM	F1-<br>tcacagagaacagattggtggatccatgccgagtccgcgccgcga<br>a<br>R1-<br>tggtggtggtggtgctcgacaagcttttaagaaccgccgagtctacc<br>cgct	2
CB2796	pET-His-SUMO-cwlM<br>∆int1	F1-<br>tcacagagaacagattggtggatccatgccgagtccgcgccgcga<br>a<br>R1-<br>tggtggtggtggtgctcgacaagcttttaagaaccgccgagtctacc<br>cgct	2
CB3166	pET-His-SUMO-cwlM<br>∆int2	F1-<br>tcacagagaacagattggtggatccatgccgagtccgcgccgcga<br>a<br>R1-<br>tggtggtggtggtgctcgacaagcttttaagaaccgccgagtctacc<br>cgct	2
CB3167	pET-His-SUMO-cwlM<br>∆int3	F1-<br>tcacagagaacagattggtggatccatgccgagtccgcgccgcga<br>a<br>R1-<br>tggtggtggtggtgctcgacaagcttttaagaaccgccgagtctacc<br>cgct	2
CB2736	pET-His-SUMO-cwlM<br>∆CT	F1-<br>tcacagagaacagattggtggatccatgccgagtccgcgccgcga<br>a<br>R1-<br>tggtggtggtggtgctcgacaagcttttacttgcctaacagatacagc<br>cgtttgaccgc	2
CB2796	pET-His-SUMO-cwlM<br>∆int1 mutation site	F1-<br>gtggctcgccacatgcgattACTAGTTCTAGTACAgtcCTA<br>agctcggggccgaagct<br>R1-<br>agcttcggccccgagctTAGgacTGTACTAGAACTAGTa<br>atcgcatgtggcgagccac	2
CB3166	pET-His-SUMO-cwlM<br>∆int2 mutation site	F1-<br>gctcgccacatgcgattcgcgaagaaTCActggtcCTTagctcg<br>gggccgaagctgtct<br>R1-<br>agacagcttcggccccgagctAAGgaccagTGAttcttcgcga<br>atcgcatgtggcgagc	2

CB3167	pET-His-SUMO-cwlM ∆int3 mutation site	F-1cgccacatgcgattcgcgaaTCAACActggtcAGTAGAtcggggccgaagctgtctggc<br>R1-gccagacagcttcggccccgaTCTACTgaccagTGTTGAttcgcgaatcgcatgtggcg	2
CB2835	pCB2835 -WT CwlM-Strep-HA	F1-ATGCTTAATTAAGAAGGAGATATACATatgatgtcgagtctgcgtcgcggtgatcgcgg<br>R1-TACTTCTCGAACTGGGGGTGGCTCCAGTCggcgccgcggccggcctgctcgaccgacaa<br>R2- HA-tag --AGCTTTCAAGCATAATCTGGTACATCATAAGGATACTTCTCGAACTGGGGGTGGCTCCA<br>R3-vector overlap -TAGGGTCCCCAATTAATTAGCTAAAGCTTTCAAGCATAATCTGGTACATCATAAGGATA	3
CB2911	pCB2911 -CwlM ∆int1-Strep-HA	F1-ATGCTTAATTAAGAAGGAGATATACATatgatgtcgagtctgcgtcgcggtgatcgcgg<br>R1-TACTTCTCGAACTGGGGGTGGCTCCAGTCggcgccgcggccggcctgctcgaccgacaa<br>R2- HA-tag --AGCTTTCAAGCATAATCTGGTACATCATAAGGATACTTCTCGAACTGGGGGTGGCTCCA<br>R3-vector overlap -TAGGGTCCCCAATTAATTAGCTAAAGCTTTCAAGCATAATCTGGTACATCATAAGGATA	3
CB2995	pCB2995 -CwlM ∆int2-Strep-HA	F1-ATGCTTAATTAAGAAGGAGATATACATatgatgtcgagtctgcgtcgcggtgatcgcgg<br>R1-TACTTCTCGAACTGGGGGTGGCTCCAGTCggcgccgcggccggcctgctcgaccgacaa<br>R2- HA-tag --AGCTTTCAAGCATAATCTGGTACATCATAAGGATACTTCTCGAACTGGGGGTGGCTCCA<br>R3-vector overlap -TAGGGTCCCCAATTAATTAGCTAAAGCTTTCAAGCATAATCTGGTACATCATAAGGATA	3
CB2996	pCB2996 -CwlM ∆int3-Strep-HA	F1-ATGCTTAATTAAGAAGGAGATATACATatgatgtcgagtctgcgtcgcggtgatcgcgg<br>R1-TACTTCTCGAACTGGGGGTGGCTCCAGTCggcgccgcggccggcctgctcgaccgacaa<br>R2- HA-tag --AGCTTTCAAGCATAATCTGGTACATCATAAGGATACTTCTCGAACTGGGGGTGGCTCCA<br>R3-vector overlap -TAGGGTCCCCAATTAATTAGCTAAAGCTTTCAAGCATAATCTGGTACATCATAAGGATA	3

CB2911	pCB2911 -CwlM Δint1-Strep-HA mutation site	F1-cgggtcgccgcacgcgatcACTTCATCTTCAACTgtgTTATCTtccggtccgcgcctg<br>R1-caggcgcggaccggaAGATAAcacAGTTGAAGATGAAGTgatcgcgtgcggcgacccg	3
CB2995	pCB2995 -CwlM Δint2-Strep-HA mutation site	F1-cgggtcgccgcacgcgatcCGAGAAGAGTCACTTgtgTTATCTtccggtccgcgcctg<br>R1-caggcgcggaccggaAGATAAcacAAGTGACTCTTCTCGgatcgcgtgcggcgacccg	3
CB2996	pCB2996 -CwlM Δint3-Strep-HA mutation site	F1-gtcgccgcacgcgatccgcgaaTCAACTctcgtgTCTcggtccggtccgcgcctgtcg<br>R1-cgacaggcgcggaccggaccgAGAcacgagAGTTGAttcgcggatcgcgtgcggcgac	3
CB3089	pUAB100-WT Tb CwlM	F1 = GAGGAATCACTTCGCAATGGCCAAGACAATTGCGGATCCgatgccgagtccgcgccgcg<br>F2 = CGGTTTCATCCCCGATCCGGAGGAATCACTTCGCAATGGCCAAGACAATTGCGGATCCg<br>R1 = ACCACCTCCAGAGCCACCGCCACCATCGATagaaccgccgagtctacccgctcgctcg<br>R2 = TCAATGGTCGAACCATTCCGGAGGACCCACCACCTCCAGAGCCACCGCCACCATCGAT	Sup.Fig. 6
CB3060	pUAB200-WT Mtb MurJ ICD	F1 = GATCCGGAGGAATCACTTCGCAATGGCCAAGACAATTGcgatggccgtgcgagcccgaa<br>R1 = ACCACCTCCAGAGCCACCGCCACCATCGATgttgcgacggcggcgggtgtaggtttc<br>R2 = ACCATGTCTACTTTACTTCCGGAGGACCCACCACCTCCAGAGCCACCGCCACCATCGAT	Sup.Fig. 6
CB3249	pUAB300-WT TbCwlM	F1 = GGTGGAGGTGGTGGGTCCGGATCCatgccgagtccgcgccgcgaagacggcgat<br>F2 = TTGAACAACCGGAATTGGGTACCGGTGGCGGTGGCGGTGGAGGTGGTGGGTCCGGATCC<br>R1 = GCCTGGCAGTCGATCGTACGCTAGTTAACttaagaaccgccgagtctacccgctc<br>R2 = CTTTCGACTGAGCCTTTCGTTTTATTTGATGCCTGGCAGTCGATCGTACGCTAGTTAAC	Sup.Fig. 6

CB3124	pUAB400-WT Mtb MurJ ICD	F1 = GTGGCGGCGGAGGTGGTGGGTCCCAATTGatggccgtgcgagcccgaatcaggagccgg<br><br>F2 = TTGAAGTCTACGAGAAGAAAGACGGTGGCGGTGGCGGCGGAGGTGGTGGGTCCCAATTG<br><br>R1 = GCAGTCGATCGTACGCTAGTTAACtcagttgcgacggcggcgggtgtaggtttcgctgt<br><br>R2 = CTTTCGACTGAGCCTTTCGTTTTATTTGATGCCTGGCAGTCGATCGTACGCTAGTTAAC	Sup.Fig. 6
CB1732	pUAB200-WT Mtb CwlM	F= GGAATCACTTCGCAATGGCCAAGACAATTGcgatgccgagtccgcgccgcgaagacggc<br>R= CACCACCTCCAGAGCCACCGCCACCATCGATagaaccgccgagtctacccgctcgctcg	Sup.Fig. 10
CB1337 (pUAB400)	pUAB400-WT Mtb CwlM	F = GAGGTGGTGGGTCCCAATTGatgccgagtccgcgccgcgaagacggc<br>R = CCTGGCAGTCGATCGTACGCTAGTTAACttaagaaccgccgagtctacccgctc	Sup.Fig. 10
CB1733	pUAB100-WT Mtb MurA	F = CTTCGCAATGGCCAAGACAATTGCGGATCCggtggccgagcgtttcgtcgtgactg<br>R = CACCTCCAGAGCCACCGCCACCATCGATacagcatacccgttcgatctcggcaccgag	Sup.Fig. 10
CB1324 (pUAB300)	pUAB300-WT Mtb MurA	F = GGTGGAGGTGGTGGGTCCGGATCCgtggccgagcgtttcgtcgtgactg<br>R = CCTGGCAGTCGATCGTACGCTAGTTAACctaacagcatacccgttcgatctc	Sup.Fig. 10

Citations

1. Abramson J, Adler J, Dunger J, Evans R, Green T, Pritzel A, Ronneberger O, Willmore L, Ballard AJ, Bambrick J, Bodenstein SW, Evans DA, Hung C-C, O'Neill M, Reiman D, Tunyasuvunakool K, Wu Z, Žemgulytė A, Arvaniti E, Beattie C, Bertolli O, Bridgland A, Cherepanov A, Congreve M, Cowen-Rivers AI, Cowie A, Figurnov M, Fuchs FB, Gladman H, Jain R, Khan YA, Low CMR, Perlin K, Potapenko A, Savy P, Singh S, Stecula A, Thillaisundaram A, Tong C, Yakneen S, Zhong ED, Zielinski M, Žídek A, Bapst V, Kohli P, Jaderberg M, Hassabis D, Jumper JM. 2024. Accurate structure prediction of biomolecular interactions with AlphaFold 3. Nature 630:493–500.

2. Singh A, Mai D, Kumar A, Steyn AJC. 2006. Dissecting virulence pathways of Mycobacterium tuberculosis through protein–protein association. Proceedings of the National Academy of Sciences 103:11346–11351.

3. Kieser KJ, Boutte CC, Kester JC, Baer CE, Barczak AK, Meniche X, Chao MC, Rego EH, Sassetti CM, Fortune SM, Rubin EJ. 2015. Phosphorylation of the Peptidoglycan Synthase PonA1 Governs the Rate of Polar Elongation in Mycobacteria. PLoS Pathog 11:e1005010.

4. Malakhov MP, Mattern MR, Malakhova OA, Drinker M, Weeks SD, Butt TR. 2004. SUMO fusions and SUMO-specific protease for efficient expression and purification of proteins. J Struct Funct Genomics 5:75–86.

5. Baer CE, Iavarone AT, Alber T, Sassetti CM. 2014. Biochemical and Spatial Coincidence in the Provisional Ser/Thr Protein Kinase Interaction Network of *Mycobacterium tuberculosis*. J Biol Chem 289:20422–20433.

6. Boutte CC, Baer CE, Papavinasasundaram K, Liu W, Chase MR, Meniche X, Fortune SM, Sassetti CM, Ioerger TR, Rubin EJ. 2016. A cytoplasmic peptidoglycan amidase homologue controls mycobacterial cell wall synthesis. eLife 5:e14590.
